# Spatial Variability of Microbial Communities and Salt Distributions Across a Latitudinal Aridity Gradient in the Atacama Desert

**DOI:** 10.1007/s00248-020-01672-w

**Published:** 2021-01-13

**Authors:** Jianxun Shen, Adam J. Wyness, Mark W. Claire, Aubrey L. Zerkle

**Affiliations:** 1 School of Earth and Environmental Sciences and Centre for Exoplanet Science, University of St Andrews, St Andrews, KY16 9AL UK; 2 Sediment Ecology Research Group, Scottish Oceans Institute, School of Biology, University of St Andrews, St Andrews, KY16 8LB UK; 3 Coastal Research Group, Department of Zoology and Entomology, Rhodes University, Grahamstown, 6139 South Africa

**Keywords:** Atacama microbiome, Function prediction, Extremophiles, Osmotic stress, Salt amendments

## Abstract

Over the past 150 million years, the Chilean Atacama Desert has been transformed into one of the most inhospitable landscapes by geophysical changes, which makes it an ideal Mars analog that has been explored for decades. However, a heavy rainfall that occurred in the Atacama in 2017 provides a unique opportunity to study the response of resident extremophiles to rapid environmental change associated with excessive water and salt shock. Here we combine mineral/salt composition measurements, amendment cell culture experiments, and next-generation sequencing analyses to study the variations in salts and microbial communities along a latitudinal aridity gradient of the Atacama Desert. In addition, we examine the reshuffling of Atacama microbiomes after the rainfall event. Analysis of microbial community composition revealed that soils within the southern arid desert were consistently dominated by Actinobacteria, Chloroflexi, Proteobacteria, Firmicutes, Bacteroidetes, Gemmatimonadetes, Planctomycetes, and Acidobacteria, and Verrucomicrobia. Intriguingly, the hyperarid microbial consortia exhibited a similar pattern to the more southern desert. Salts at the shallow subsurface were dissolved and leached down to a deeper layer, challenging indigenous microorganisms with the increasing osmotic stress. Microbial viability was found to change with aridity and rainfall events. This study sheds light on the structure of xerotolerant, halotolerant, and radioresistant microbiomes from the hyperarid northern desert to the less arid southern transition region, as well as their response to changes in water availability.

**Supplementary Information:**

The online version contains supplementary material available at 10.1007/s00248-020-01672-w.

## Introduction

The Atacama Desert in northern Chile is the driest non-polar terrestrial desert on Earth, spanning more than 1000 km in length from central Chile to southern Peru [1]. It is widely accepted as a Mars analog system based on its similar geomorphic landscape to Mars and multiple physicochemical aspects such as its hyperaridity, absence of water weathering, high ultraviolet (UV) radiation, low levels of organic carbon, and large reservoirs of oxidants [2]. The microbial life is unique from other terrestrial locations because these organisms have been exposed to extreme conditions since the Late Jurassic 150 million years ago [1]. Thus, the environmental gradients within the Atacama Desert serve as an excellent model to investigate the influence of long-term aridity and different frequencies of precipitation-led water stress on soil microbial communities.

The newly available Atacama Database of microbiology [3] records 2302 microorganisms in the Atacama Desert, with reference to 633 previously published papers between 1966 and 2016. Among these microorganisms, 1741 species are from the Domain Bacteria. However, bacteria from unknown phyla still comprise 40% of the currently recorded Atacama microbiome [3]. These microbes are distributed throughout the soil profile, with surface inhabitants are generally more tolerant to strong UV radiation, and those found deeper in soils are more tolerant to hypersaline conditions [4]. Despite the extreme aridity, desiccation may not be the sole or even the primary factor influencing microbial life in desert environments, and highly saline and oxidizing soils can also be crucial factors.

Counter-intuitively, an abrupt increase in water availability in hyperarid soils is extremely harmful to xerotolerant microorganisms because these cells are induced to transform from the defensive or dormant state to the metabolically active state while unexpectedly being exposed to attack from extreme temperature and UV radiation [5]. In addition, excessive water causes high osmotic shock to the microbial semipermeable membrane and disturbs their survival strategies adapted for limited moisture [6, 7]. After the heavy rainfall event in 2017 at the core region of the Atacama Desert, 75–87% of pre-rainfall species were undetectable, and no viable archaea or eukaryotes were detected in undrained brines [6]. Although these rainfall events can severely damage the extremotolerant microbial communities, previous studies demonstrated that the community structure can recover after 1 month in the central Namib Desert [8], or for more than 1 year at Salar Grande in the northern Atacama Desert [9], using a variety of biochemical mechanisms and osmoregulatory systems. Immediately after rainfall, microorganisms start producing proteins and metabolites that are crucial in fundamental biosynthetic pathways, energy supplements, desiccation resistance, radiation protection, and oxidation defense for the preparation of the upcoming hyperarid period [10].

Martian surface is likely to have been much wetter around 3–4 billion years ago [11, 12] supported by the evidence of carved outflow channels, valleys, evaporitic basins, and northern paleoceanographic landforms [13–15]. Due to the biochemical importance of water, potentially extant microorganisms may have been thrived on early Mars. Between then and the drier planet we recognize today, a transitional dry period with occasional moisture has occurred [16]. On the more recent dry Mars, the soil pH is slightly alkaline (7.7–8.3) as determined by the Phoenix rover [17], which is similar to the pH 6.6–9.2 of Atacama soils [18–21]. Another similarity between Martian and Atacama soils is the nearly equivalent concentrations of highly soluble perchlorate (0.4–0.6% for Mars [22] and 0.5–0.6% for Atacama [23]) and total nitrogen (0.007–0.11% for Mars [24] and 0.01–0.15% for Atacama [25]). Although precipitation is absent on the modern Mars, a radar analysis validates the existence of liquid water trapped by polar ice [26], and detection results by the Curiosity rover show evidence of temporary subsurface liquid during nighttime on equatorial Mars and possibly beyond [27]. Therefore, the rare heavy rain in the hyperarid Atacama acts as an analog to the transient availability of liquid water on Mars. Abundant hygroscopic chloride minerals and hydrated sulfate minerals were detected in the Phoenix and Curiosity exploration locations [28–30].

This study investigated the differences in microbiome and soil salt compositions along a latitudinal precipitation gradient of the Atacama Desert after the heavy rainfall event. Based on the soil relative humidity, Azua-Bustos et al. (2015) argued that María Elena South (MES) was drier than the commonly known hyperarid Yungay region [31], but no next-generation sequencing has been conducted to examine the MES microbial community. The primary research goals of this study are to explore the microbial community structure and functions in MES and their changes toward the more humid sites, to compare with some previous pre-rainfall Atacama Desert studies, and to investigate how water and salt amendments affect microbial growth in Atacama soils. Since the sampling was conducted half a year after the most recent massive rainfall, the recovery of microbial communities in the hyperarid core area was hypothesized to be in some middle point between the pre-rainfall condition and the more humid condition. The hyperarid sites that received more precipitation were hypothesized to be decimated more seriously and thus might have lower biomass. As high salinity results in hyperosmotic pressure, excessive salt amendments were hypothesized to decrease the microbial abundance. Considering the hygroscopic property of some abundant Atacama minerals such as halite (NaCl) and gypsum (CaSO_4_·2H_2_O), previous researchers argued that these minerals could be ideal microhabitats for endolithic microorganisms within arid environments due to the moisture support [32–35]. According to a water-stable isotope study [36], the crystallization water of gypsum could supply > 70% water source to even higher plants. Despite the hypersaline condition, chloride and sulfate were hypothesized to benefit the microbial survival and growth to some extent. To live under long-term arid conditions and tackle the rewetting after rainfall, microorganisms hypothetically possessed pathways to resist osmotic stress, ionizing radiation, extreme dryness, and to utilize limited nutrients. Marker gene metabolic inference analysis was performed to preliminarily examine this postulation.

## Methods

### Site descriptions and soil characterizations

**Fig. 1:**
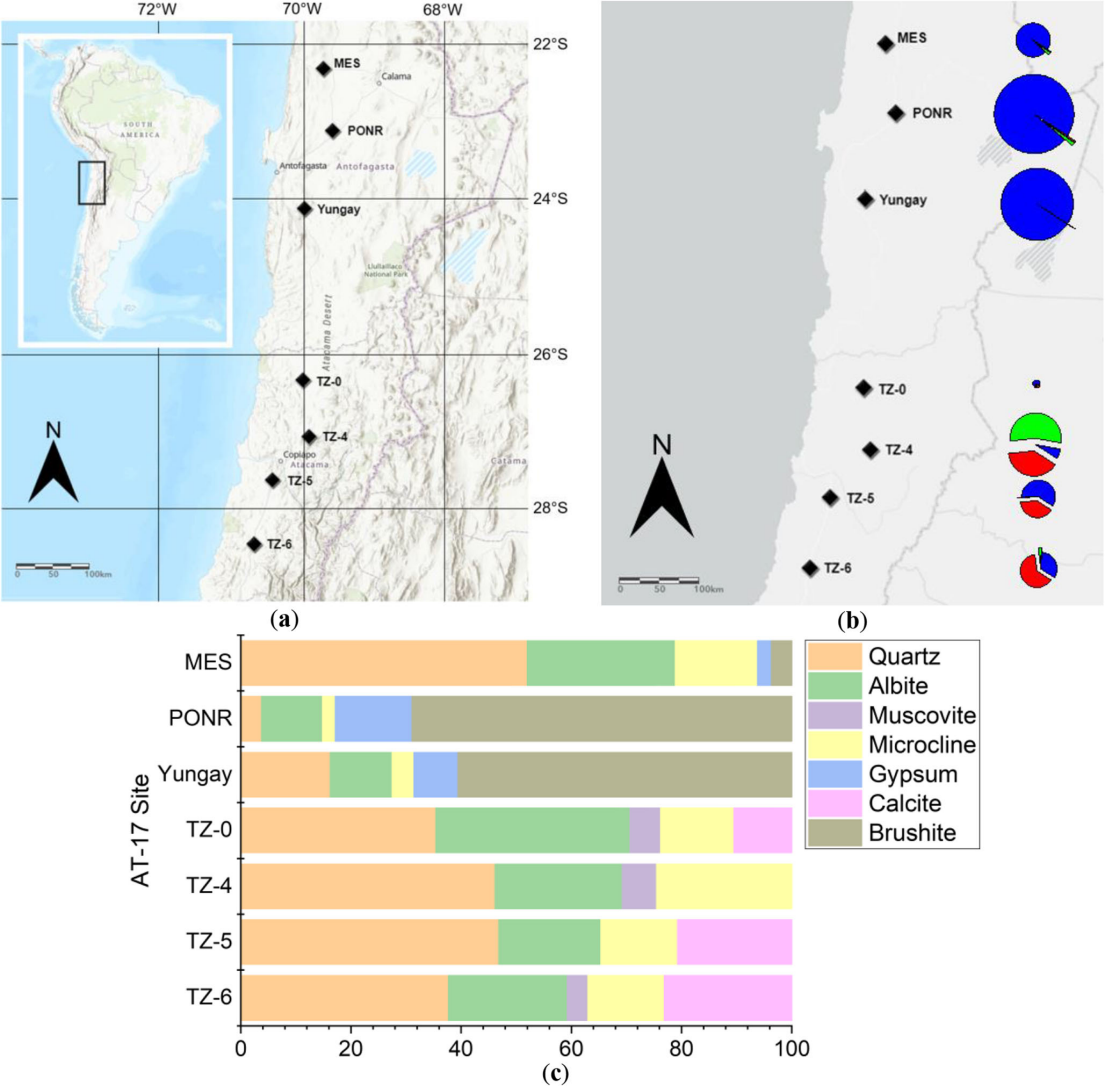
**a** Geographic locations of the Atacama Desert AT-17 sampling sites, including María Elena South (MES), PONR (Point of No Return), Yungay, transition zone 0 (TZ-0), TZ-4, TZ-5, and TZ-6. **b** Soil collection sites in the Atacama Desert, illustrating the major salt distribution of different sites. The size of the pie charts represents the concentrations of soluble salts (Cl^−^, red; NO_3_^−^, green; SO_4_^2−^, blue). The areas of pie charts range from 30 to 17,000 ppm. **c** Percentages of different minerals in AT-17 samples determined by X-ray diffraction (XRD)

Samples were collected from the Atacama Desert, northern Chile, on November 30–December 6, 2017, after an unprecedented heavy rainfall event that expanded from the Yungay region during June 6–7 in 2017 [6] with 19.6 mm of rainfall, according to the record at Antofagasta Rain Gauge (23.5975° S, 70.3867° W, altitude: 50 m). The rainfall at each sampled site, for June 2017, was listed in Table S1. Soils were collected from seven field sites along a ~ 800 km north to south latitudinal transect from 22° S to 29° S (Fig. 1a and Table S1) as described by Shen et al. (2019). Briefly, samples were obtained from three hyperarid sites (María Elena South (MES), Point of No Return (PONR), and Yungay) and four-transition zone sites (TZ-0, TZ-4, TZ-5, and TZ-6). Visible vegetation cover (sparse dried grasses and shrubs) appeared in regions close to the sampling sites of TZ-4, TZ-5, and TZ-6. Within each site, three random pits were sampled at a depth of 10–20 cm for geochemical analyses and DNA extractions, and the third sampling pit was additionally sampled for microbial cell counting and cultivations. All samples were collected with a sterile sampling trowel, placed into sterile Whirl-Pak® bags (Nasco, Fort Atkinson, USA). Soils obtained in 2017 were collectively referred to as AT-17.

General soil properties (pH, electrical conductivity, major sediment elements by X-ray fluorescence (XRF) methods, and the concentrations of total organic carbon (TOC), total organic nitrogen (TON), carbonate, and nitrate) of AT-17 geochemical samples were reported in Shen et al. (2019). AT-17 geochemical samples were sieved through a 1.4-mm sieve prior to ion chromatography for soluble anion determinations as described in Shen et al. (2019).

Additionally, AT-17 soil samples for X-ray diffraction (XRD) were crushed using a Planetary Micro Mill (PULVERISETTE 6, FRITSCH) and sieved through a 355-μm sieve. The mineralogy of these crushed and sieved samples was analyzed on a Philips X-Ray Diffractometer (PW1830 generator, PW1050/80 goniometer, PW1710 diffractometer) using Co Kα radiation. Generator settings for the measurement were 30 kV, 30 mA, 3–70° range 2θ scan, 0.01° step, and 1 s/step. Mineral phases were identified using the EVA 2 software from SOCABIM with the ICDD PDF-2 database.

### DNA extractions and sequencing

Due to the low biomass in Atacama soils, AT-17 microbiological samples for DNA extraction were well sealed and stored under dry conditions (30–70% relative humidity) for 1 year at 4 °C prior to finding a suitable cell lysis device, the Precellys 24 tissue homogenizer. The daily atmospheric relative humidity of the Atacama Desert ranges from 0% at noon to 100% at midnight in the hyperarid core area, with a mean value of ~ 30% [31, 37, 38]. Since warmer air has a higher capacity for moisture, the moisture content at 4 °C is generally similar to that of the air above Atacama soils. The annual temperature of the Atacama Desert was between − 5 °C and 40 °C [25, 39], so we attempted to store soils at a low but comparable temperature to keep microbial communities alive but as dormant as possible. Due to the extreme aridness of these dry sandy soil samples, the effect of the storage duration should be insignificant, as even biolipids could preserve for more than one billion years [40]. DNA of AT-17 soils from three pits of each site were extracted together with an extraction blank using the MP Biomedicals™ FastDNA™ SPIN Kit for Soil following a modified manufacturer’s protocol: during the cell lysis step, mixtures were incubated at room temperature for 1 h. DNA extracts were amplified in triplicate for barcoded Illumina 16S metagenomic sequencing using KAPA HiFi HotStart ReadyMix (KAPA Biosystems, Roche, UK) and 16S rRNA primer pair 341F, 5′-CCTACGGGNGGCWGCAG-3′, and 785R, 5′-GACTACHVGGGTATCTAATCC-3′ using the Nextera index kit (Illumina®). Amplicons were quantified using an Invitrogen™ Qubit™ 3.0 Fluorometer, and only samples with more than about 0.5 ng/μL yields (i.e., 3 pits in MES, 2 pits in TZ-0, 3 pits in TZ-4, 3 pits in TZ-5, and 3 pits in TZ-6) were passed for following preparation of sequencing. Triplicate amplicons from each pit were pooled together and concentrated by heating at 50 °C to a final volume of 50 μl. 16S rRNA amplicons for Illumina MiSeq System was replicated and sequenced by following *16S Metagenomic Sequencing Library Preparation* together with a 20% PhiX control and the extraction blank using paired-end 300-bp reads with v3 Chemistry, modified by extending the amplicon PCR reactions from 25 to 29 cycles due to low product yield.

### Assessments of bacterial abundance and viability

The abundance and viability (proportion of viable cells in total cells) of AT-17 microbial communities were analyzed via duplicate trypan blue staining assay within 1 month of sample collection and cultivation methods within 1 month and replicate within half a year after sample collection. Duplicate AT-17 microbiological soils from the third sampling pit of each site were suspended and 10× serially diluted two to four times to clear out any sand particles. 0.4% Trypan blue was added to 9× of the dilute solution. Viable (non-colored) and non-viable (blue) microbial cells were mounted on a Hirschmann Instruments™ Counting Chamber and counted using oil immersion light microscopy (AmScope).

Duplicate AT-17 samples from the third sampling pit of each site were suspended and homogenized in 1:1 volume (mL):weight (g) of sterilized ultrapure water for microbial cultivations. Different nutrient effects from various cell culture plates on the colony growth of culturable heterotrophic microbes were considered, so four types of agar plates were selected in this culturing experiment: (1) ultrapure agarose plate (15 g/L agarose), with agarose as the sole nutrient source; (2) tryptic soy agar plate (15 g/L agar, 15 g/L peptone, 5 g/L soyabean digest, 0.7 g/L lecithin, 5 g/L Tween 80), with casein and plant-derived organics and glycerophospholipids as additional nutrients; (3) Luria-Bertani (LB) agar plate (15 g/L agar, 10 g/L tryptone, 5 g/L yeast extract, 5 g/L NaCl), with casein and yeast-derived organics and table salt as additional nutrients; and (4) plate count agar (9 g/L agar, 5 g/L tryptone, 2.5 g/L yeast extract, 1 g/L dextrose), with casein and yeast-derived organics and glucose as additional nutrients. Based on some preliminary tests for countable colony number estimations, an appropriate amount of soil suspension was spread on these four types of culture medium. Visible colonies were counted after 20 days of incubation at 21 °C. Without additional amendments, these sets were also the controls for colony number normalizations described in Table 1.

**Table 1:** Results of cell cultures on ultrapure agarose, tryptic soy agar, LB agar, and plate count agar plates with water amendments, recording the change in the order of magnitude of CFUs with water amendments (all numbers are scaled by logarithmic transformation and normalized as the difference from the plates without amendments). Positive effects of different volumes of water on microbial growth are in italics, and those caused increase in CFUs more than 1 and 2 orders of magnitude are labeled with ^+^ and ^++^, respectively

Type of culture plate	Amendment	MES	PONR	Yungay	TZ-0	TZ-4	TZ-5	TZ-6
Ultrapure agarose	1.5 mL H_2_O	− 1.18	*0.05*	*0.55*	− 0.89	− 0.32	− 1.15	− 1.08
	3 mL H_2_O	− 1.18	*0.41*	*0.39*	− 0.60	− 0.27	− 0.97	− 0.63
	4.5 mL H_2_O	− 1.18	− 1.11	*0.29*	− 0.84	− 0.21	− 1.05	− 0.90
Tryptic soy agar	1.5 mL H_2_O	*1.15*^*+*^	*0.42*	− 0.58	− 0.24	− 0.17	− 0.36	− 0.16
	3 mL H_2_O	*0.05*	− 0.19	*1.57*^*+*^	− 0.25	− 0.30	− 0.05	*0.13*
	4.5 mL H_2_O	*2.20*^*++*^	*1.80*^*+*^	*1.83*^*+*^	− 0.22	*1.01*^*+*^	− 0.41	*0.20*
LB agar	1.5 mL H_2_O	*0.63*	*0.29*	− 0.41	*0.19*	− 1.47	− 0.21	*0.49*
	3 mL H_2_O	*0.34*	*0.53*	*2.36*^*++*^	*0.42*	− 0.95	*0.22*	*0.32*
	4.5 mL H_2_O	− 0.36	*2.46*^*++*^	*2.06*^*++*^	*0.73*	*0.71*	− 0.40	*0.45*
Plate count agar	1.5 mL H_2_O	*0.65*	*1.65*^*+*^	*0.02*	− 0.65	− 0.33	*0.51*	*0.71*
	3 mL H_2_O	*1.10*^*+*^	*0.13*	*0.75*	− 0.76	− 0.41	*0.16*	− 0.32
	4.5 mL H_2_O	*0.91*	*2.65*^*++*^	*0.94*	− 0.34	*0.29*	*0.84*	*0.52*

### Salt and water amendments

To inspect the effects of a variety of excessive dissolved salts on the viable microbial community, soils from the third sampling pit of each site were amended with 4.5 mL salt solutions. Solutions of 10% sodium chloride, 10% sodium sulfate, 10% sodium carbonate, 10% sodium acetate, and 10% sodium l-lactate were prepared in ultrapure water and autoclaved at 121 °C for 30 min. After cooling to room temperature, 10 g of AT-17 soil was combined with the salt solution for 4 days at 21 °C [41]. Duplicate salt-amended soils were suspended in an appropriate volume of sterile ultrapure water and spread on ultrapure agarose, tryptic soy agar, LB agar, and plate count agar plates. Plates were sealed with Bemis™ Parafilm™ M Laboratory Wrapping Film and incubated for 20 days at 21 °C prior to cell counting [42]. Colony-forming units (CFUs) were determined by the multiplication of the number of colonies, dilution factor, and 1.45 to account for the addition of 4.5 mL solution to 10 g soil.

In addition, 1.5 mL, 3 mL, and 4.5 mL of sterile ultrapure water were added to each 10 g of AT-17 microbiological samples, which covers about the 1/3, 2/3, and full of soil area, respectively. Since the bottom diameter of Petri dishes used for culturing experiments is 90 mm, these volumes of water are equivalent to approximately 0.24, 0.47, and 0.71 mm precipitation, respectively. CFUs were cultured and determined in the same manner as salt amendments. CFUs from cultivation experiments were converted via common logarithmic transformation. CFUs on salt amendments were standardized by subtracting their respective 4.5-mL water amendment groups; and CFUs on water amendments were standardized by subtracting their corresponding groups without any amendments.

### Data analyses

Illumina sequence reads of AT-17 bacterial sequencing results were processed with the open-source program Quantitative Insights into Microbial Ecology 2 (QIIME 2). Reads were combined and demultiplexed into Casava 1.8 paired-end format. Reads of low quality were denoised with the DADA2 pipeline plugin of QIIME 2. Reads were filtered by trimming the first 13 base pairs and removing those of low quality, with ambiguous characters and missing barcoded primers, of a length less than 300 base pairs. AT-17 sequences were then clustered into operational taxonomic units (OTUs) at a 99% similarity cutoff using VSEARCH de novo clustering. The quality of total clustered sequences was double confirmed via noise and chimera pattern checking by VSEARCH and UCHIME. The representative taxonomic identities were aligned at 99% full-length sequence homology using a pre-trained naïve Bayesian classifier [43] based on the SILVA 132 marker gene reference database. All mitochondrial and chloroplast reads were removed using the taxonomy-based filtering of tables and sequences method.

OTU tables were rarefied to remove sampling depth heterogeneity at 23,384 even sampling depth. Alpha diversity indices (binary logarithmic Shannon diversity, Faith’s phylogenetic diversity, Pielou’s Evenness, and observed OTUs) were computed with the alpha-phylogenetic package of QIIME 2. Alpha rarefaction curves were generated at the maximum 20,000 sequencing depth. Characteristic bacterial phyla associated with the sampling year and aridity were predicted and clustered with the q2-sample-classifier package showing the 100 most representative sequences. Analysis of the composition of microbiomes (ANCOM) was employed to determine the genera that were significantly different among sampling year and aridity. Hierarchical clustering of each individual site using the Bray-Curtis distance matrix was performed in Past 4.03.

Enzyme prediction and functional annotation of constructed sequences were performed in software Phylogenetic Investigation of Communities by Reconstruction of Unobserved States 2 (PICRUSt2) based on marker gene sequences. Representative OTUs with nearest-sequenced taxon index (NSTI) more than 2 were excluded from the output. The abundance of predicted functional pathways was standardized as the percentage of the sum of read depths. MetaCyc pathway identifiers were linked to their respective functions at the secondary superclass level using the MetaCyc pathway hierarchy system.

## Results

### Soil geochemical features

To investigate the Atacama soil physicochemical context, samples were analyzed by total organic C and N (TOC and TON) quantifications, X-ray diffraction (XRD), X-ray fluorescence (XRF), and ion chromatography (IC). AT-17 sites were generally characterized by quartz (44 ± 6%), albite (25 ± 6%), and microcline (16 ± 4%). However, hydrated minerals, brushite and gypsum, took over a significant percentage (65 ± 4% and 11 ± 3% respectively) in the two post-rainfall hyperarid sites—PONR and Yungay (Fig. 1c and Figure S1). The AT-17 soluble salts (i.e., chloride, nitrate, and sulfate) varied significantly between samples: chloride concentrations ranged from 5.8 parts per million (ppm) in TZ-0 to 3600 ppm in TZ-4; nitrate ranged from 1.9 ppm in TZ-0 to 5000 ppm in TZ-4; and sulfate ranged from 24 ppm in TZ-0 to 17,000 ppm in PONR (Table S2). Total soluble salts remained at about the same level within PONR and Yungay and within MES, TZ-5, and TZ-6 (Fig. 1b).

### Bacterial abundance and viability

**Fig. 2:**
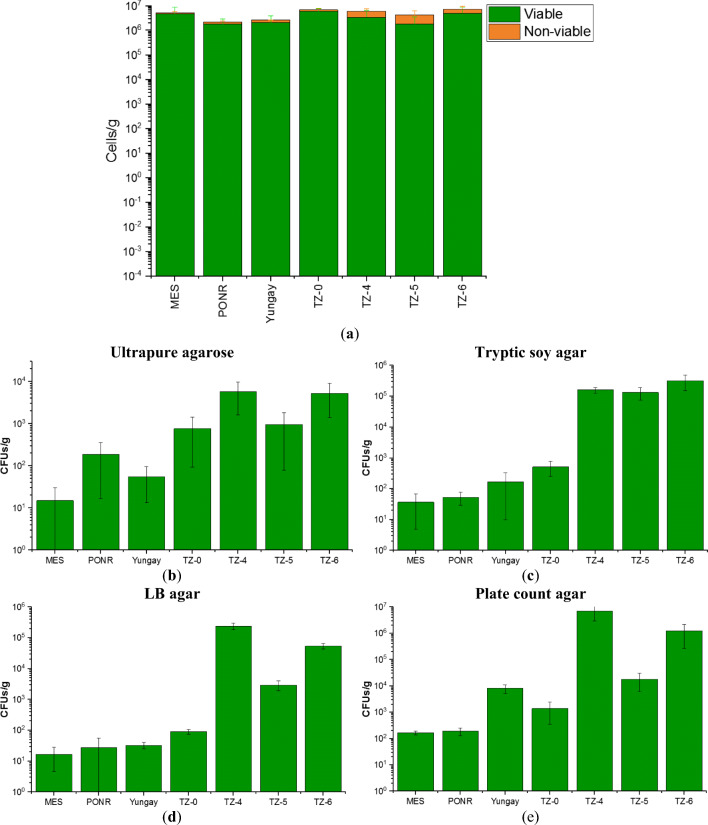
**a** Cumulative bacterial cell counts per gram of soils of AT-17 samples determined by trypan blue staining assay analyses and viable aerobic heterotrophic colony-forming units (CFUs) on **b** ultrapure agarose, **c** tryptic soy agar, **d** LB agar, and **e** plate count agar plates

To assess the abundance and viability of bacterial communities, trypan blue staining assay and cell culture experiments were employed. The viability hereafter was quantified by the ratios of viable cells to total cell counts. Trypan blue staining-determined viable and total cell counts of hyperarid sites PONR and Yungay were lower than the southern transition sites (Fig. 2a and Table S3). However, no significant difference in the viable and total cell counts was noted between the northernmost hyperarid site MES and southern transition sites. The active culturable colonies on different types of culture plates increased appreciably toward the transition zone with higher annual precipitation (Fig. 2b, c, d, e). In general, TZ-5 had lower microbial abundance than TZ-4 and TZ-6. The viable:non-viable ratios in AT-17 soils varied from 0.7 in TZ-5 to 8.0 in MES (Table S3), and these ratios were generally higher in the dryer sites than the more humid sites.

**Fig. 3:**
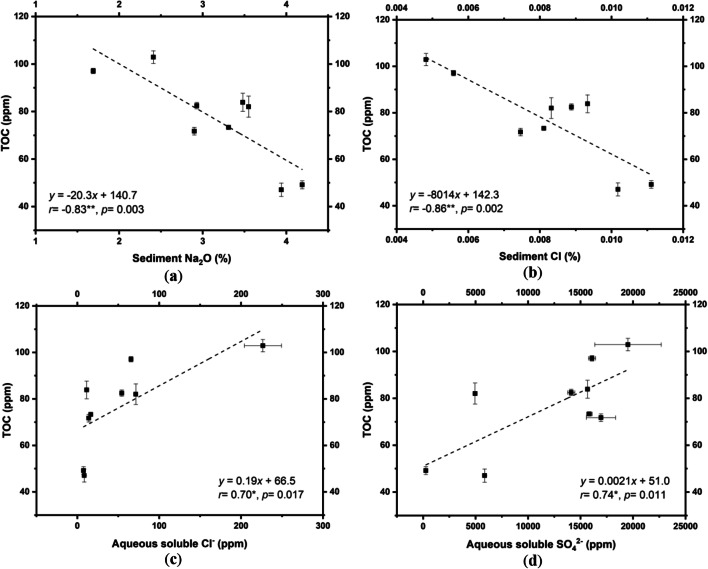
Plots of total organic carbon (TOC) and **a** sediment sodium, **b** sediment chloride, **c** water-soluble chloride, and **d** water-soluble sulfate in hyperarid sites

Within the hyperarid core of the Atacama Desert in 2017, TOC was negatively correlated with the contents of sediment sodium (Na_2_O) and chloride (Cl) (Fig. 3a and b). However, TOC was positively associated with the concentrations of the water-soluble chloride (Cl) and sulfate (SO_4_) (Fig. 3c and d).

Soil microbial communities showed different preferences to the water volume on different agar plates. Water amendment cultures (1.5, 3, and 4.5 mL) demonstrated that transition sites were slightly less water limited than hyperarid sites because increasing water content did not change CFUs (Table 1 and Table S4). In general, an increase in water content from 0 to 4.5 mL increased bacterial cell counts, but the growth rates did not always increase with further water addition. No CFU decrease in the water amendments was more than 1.5 orders of magnitude (Table 1). Overall, excessive sodium chloride and sodium carbonate amendments decreased cell counts; sodium sulfate and sodium acetate had no effect; and sodium l-lactate increased cell counts (Table 2 and Table S4). Sites with higher annual precipitation generally had a higher tolerance or higher preference to the amendments with these salts, except for sodium carbonate (Table 2).

**Table 2:** Results of cell cultures on ultrapure agarose, tryptic soy agar, LB agar, and plate count agar plates with salt amendments, recording the change in the order of magnitude of CFUs with salt amendments (all numbers are scaled by logarithmic transformation and normalized as the difference from the plates with 4.5-mL water amendments). Negative effects of different salts on microbial growth are in italics, and those caused the decrease in CFUs more than 1, 2, 3, and 4 orders of magnitude are labeled with ^-^, ^--^, ^---^, and ^----^, respectively

Type of culture plate	Amendment	MES	PONR	Yungay	TZ-0	TZ-4	TZ-5	TZ-6
Ultrapure agarose	Chloride	0	− *0.78*	0.64	0.42	− *0.04*	1.67	0.64
	Sulfate	0	− *1.16*^*-*^	− *1.64*^*-*^	− *0.62*	− *0.35*	0	0.06
	Carbonate	0	− *0.48*	− *0.01*	− *2.05*^*--*^	− *0.48*	0	0.72
	Acetate	0	− *1.16*^*-*^	0.37	0.64	− *0.94*	0.90	0.35
	l-lactate	2.34	1.87	0.67	1.00	− *1.49*^*-*^	1.63	0.18
Tryptic soy agar	Chloride	− *2.90*^*--*^	− *2.45*^*--*^	0.50	− *0.24*	− *1.59*^*-*^	1.22	0.01
	Sulfate	− *2.42*^*--*^	− *2.85*^*--*^	0.05	0.58	− *0.77*	0.82	0.01
	Carbonate	− *1.16*^*-*^	− *2.54*^*--*^	0.38	− *1.63*^*-*^	− *3.74*^*---*^	0.02	− *0.14*
	Acetate	− *3.38*^*---*^	− *3.15*^*---*^	− *2.09*^*--*^	− *0.07*	− *0.51*	0.44	0.34
	l-lactate	0.58	− *1.77*^*-*^	− *0.21*	0.27	0.03	0.20	− *0.03*
LB agar	Chloride	− *0.86*	− *3.52*^*---*^	− *1.23*^*-*^	− *0.56*	− *2.61*^*--*^	− *0.08*	− *0.11*
	Sulfate	0.48	− *3.52*^*---*^	− *1.41*^*-*^	0.24	− *2.12*^*--*^	0.40	0.38
	Carbonate	2.57	− *2.74*^*--*^	− *1.84*^*-*^	− *2.69*^*--*^	− *3.60*^*---*^	0.22	− *1.54*^-^
	Acetate	0.12	− *3.22*^*---*^	− *1.18*^*-*^	− *0.14*	− *0.35*	0.57	0.59
	l-lactate	1.35	− *1.64*^*-*^	0.47	0.34	0.43	0.72	0.44
Plate count agar	Chloride	− *2.75*^*--*^	− *3.21*^*---*^	− *3.38*^*---*^	− *0.43*	− *0.85*	− *0.33*	− *0.86*
	Sulfate	− *1.16*^*-*^	− *1.99*^*-*^	− *4.84*^*----*^	1.16	− *0.34*	− *0.33*	− *0.99*
	Carbonate	0.07	− *2.69*^*--*^	− *2.60*^*--*^	− *1.08*^*-*^	− *3.49*^*---*^	− *0.29*	− *1.37*^*-*^
	Acetate	− *2.75*^*--*^	− *3.65*^*---*^	− *2.71*^*--*^	− *0.52*	− *0.70*	− *0.73*	− *0.49*
	l-lactate	0.33	− *2.46*^*--*^	− *0.01*	0.65	0.32	0.50	− *0.20*

### Microbial community diversity and pathway analysis

To investigate the impact of aridity on Atacama microbial communities, we performed a soil metagenomic analysis of AT-17 bacterial sequence data. The extracted DNA concentrations of all samples were below the limit of detection of Qubit 3.0 dsDNA high-sensitivity assay before amplification. After 29 cycles of PCR, the amplicon concentrations of all samples were exhibited in Table 3. Unfortunately, since the DNA concentrations extracted from PONR and Yungay were not sufficient for sequencing, we could not compare them. For the remaining samples, biodiversity was the lowest in MES, while the highest in TZ-5 and TZ-6 (Table 3 and Figure S2). The diversity was slightly higher in TZ-0 than TZ-4.

**Table 3:** Concentrations of triplicate amplicons (means ± standard errors), Shannon diversity, Faith’s phylogenetic diversity (PD), species evenness, and observed OTU richness indices of AT-17 samples

Site	Amplicon (ng/μL)	Shannon (binary log)	Faith’s PD	Evenness	Observed OTUs
AT17-M1 (MES pit 1)	0.56 ± 0.05	6.73	25.3	0.833	272
AT17-M2 (MES pit 2)	0.56 ± 0.06	5.59	16.8	0.767	156
AT17-M3 (MES pit 3)	0.47 ± 0.35	5.60	18.9	0.772	152
AT17-P1 (PONR pit 1)	0.05 ± 0.02	-	-	-	-
AT17-P2 (PONR pit 2)	0.03 ± 0.03	-	-	-	-
AT17-P3 (PONR pit 3)	0.04 ± 0.00	-	-	-	-
AT17-Y1 (Yungay pit 1)	0.04 ± 0.00	-	-	-	-
AT17-Y2 (Yungay pit 2)	< 0.01	-	-	-	-
AT17-Y3 (Yungay pit 3)	0.05 ± 0.05	-	-	-	-
AT17-T01 (TZ-0 pit 1)	35.8 ± 7.7	7.59	43.9	0.799	722
AT17-T02 (TZ-0 pit 2)	18.6 ± 3.4	6.99	37.6	0.767	550
AT17-T41 (TZ-4 pit 1)	10.9 ± 0.8	7.09	38.6	0.793	494
AT17-T42 (TZ-4 pit 2)	7.3 ± 1.3	7.11	38.4	0.787	522
AT17-T43 (TZ-4 pit 3)	38.3 ± 8.2	6.72	33.1	0.788	371
AT17-T51 (TZ-5 pit 1)	35.1 ± 8.5	8.43	50.6	0.892	699
AT17-T52 (TZ-5 pit 2)	52.2 ± 5.0	8.49	73.6	0.833	1172
AT17-T53 (TZ-5 pit 3)	47.9 ± 7.2	7.84	49.9	0.842	632
AT17-T61 (TZ-6 pit 1)	71.7 ± 8.1	8.32	53.0	0.859	821
AT17-T62 (TZ-6 pit 2)	42.7 ± 12.4	8.50	52.8	0.879	813
AT17-T63 (TZ-6 pit 3)	58.7 ± 2.5	8.60	52.8	0.889	815

**Fig. 4:**
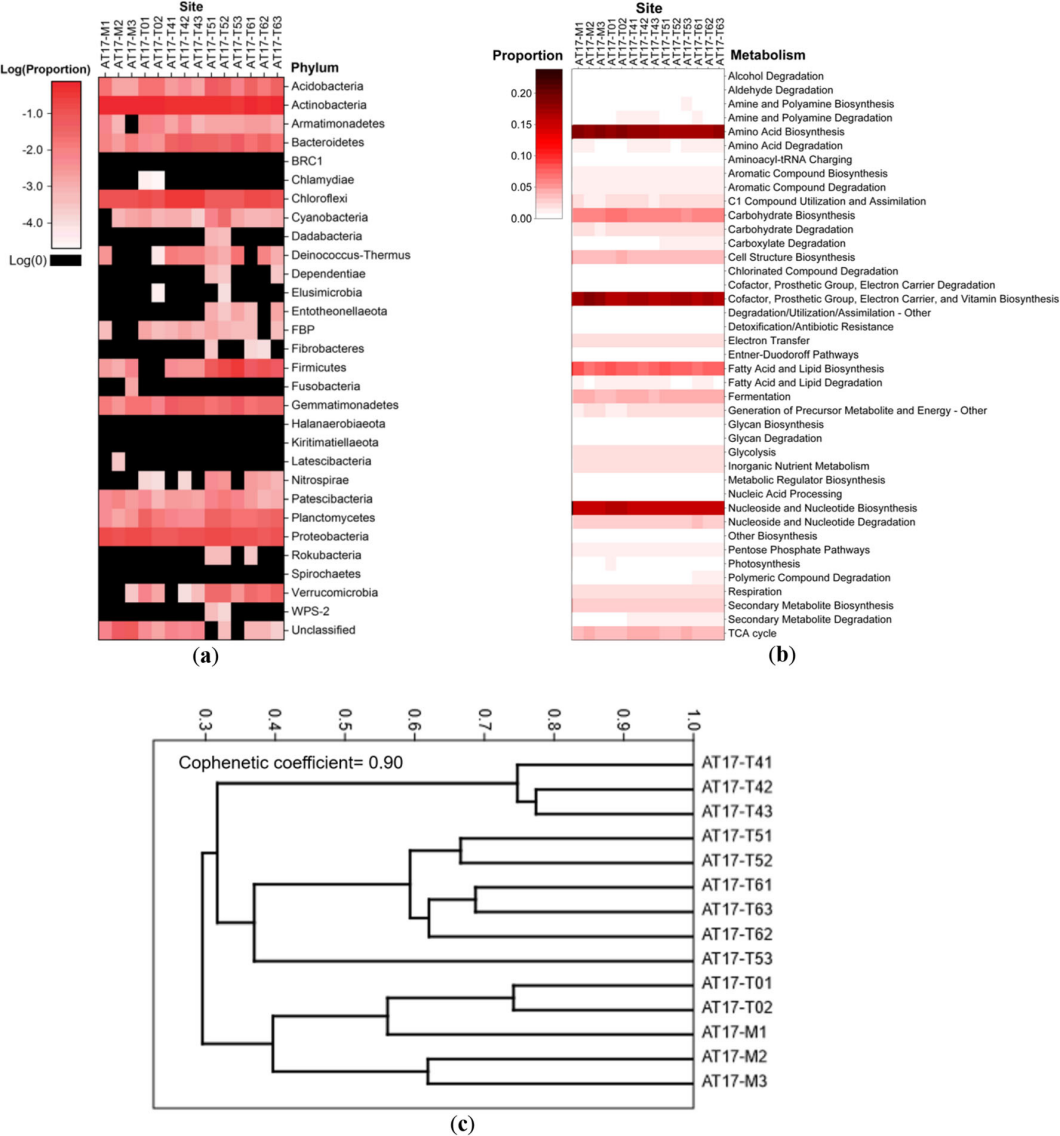
**a** The heatmap illustrating phylum level microbial composition on logarithmic scales in Atacama soils from AT-17 sequencing data. **b** Relative abundance of predicted microbial metabolisms presented as the secondary superclass in MetaCyc classification system. **c** Hierarchical clustering (Bray-Curtis distance matrix and unweighted pair group method with arithmetic mean algorithm) of microbial compositions at the species level in AT-17 sites. Abbreviations as in Table 3

In total, 464 bacterial families, 832 genera, and 1112 species were determined. Considering their large quantity, we plotted at the phylum level only (Fig. 4a) and listed the named families with > 2% proportion in at least one sequenced sample (Table 4). The bacterial phyla dominated soils within the AT-17 hyperarid MES were Actinobacteria (66.27 ± 3.94%), Proteobacteria (12.65 ± 2.31%), and Chloroflexi (11.83 ± 0.79%); and those dominated soils within AT-17 arid southern desert were similarly Actinobacteria (50.42 ± 11.84%), Chloroflexi (16.14 ± 8.27%), and Proteobacteria (11.47 ± 2.92%) (Fig. 4a).

**Table 4:** Compositions of bacterial families in sampling pits of each Atacama site, displaying the named families that were more than 2% abundance (percentages in italics) in at least one pit sample

Phylum	Family (%)	M1	M2	M3	T01	T02	T41	T42	T43	T51	T52	T53	T61	T62	T63
Actinobacteria	*Geodermatophilaceae*	0.6	*2.1*	*2.0*	0.2	0.1	*5.6*	*6.5*	*5.6*	*2.4*	1.0	1.4	*2.4*	*2.6*	*2.2*
	*Nocardioidaceae*	0.5	0.1	0.1	1.0	0.5	*2.1*	2.0	1.8	0.8	1.2	0.2	1.1	1.5	1.9
	*Pseudonocardiaceae*	0.2	0.5	0.0	0.6	0.5	*2.5*	1.9	*3.2*	2.0	1.8	0.2	*10.2*	*4.3*	1.8
	*Euzebyaceae*	1.6	1.3	1.1	1.1	0.9	*4.7*	*3.4*	*3.9*	1.1	1.4	*3.5*	1.5	*3.9*	*3.0*
	*Nitriliruptoraceae*	*2.0*	*3.8*	0.0	*3.5*	*10.9*	0.7	0.6	0.3	0.4	0.5	*4.3*	0.6	1.3	0.2
	*Rubrobacteriaceae*	*5.3*	*9.0*	*12.3*	*8.1*	*9.2*	*9.6*	*4.6*	*8.9*	*10.8*	*5.2*	*4.7*	*9.1*	*11.8*	*8.0*
	*Solirubrobacteraceae*	1.7	1.7	0.9	*3.8*	*3.7*	*7.5*	*11.8*	*10.9*	*3.1*	*2.5*	*3.2*	*3.4*	*4.4*	*2.4*
Bacteroidetes	*Hymenobacteraceae*	0.0	0.0	0.0	0.0	0.0	*3.0*	*3.7*	*2.9*	0.7	0.2	0.6	0.8	0.3	0.3
Chloroflexi	*AKIW781*	*3.7*	*6.2*	*2.6*	*3.5*	1.7	*9.5*	*13.8*	*10.9*	*3.4*	*3.5*	1.6	*5.4*	*5.3*	*2.0*
	*Kallotenuaceae*	0.0	0.0	0.0	0.0	0.0	*5.6*	0.8	1.8	0.0	0.0	0.0	0.2	0.2	0.1
	*Thermobaculaceae*	*2.1*	*2.3*	*2.3*	1.1	1.4	*3.1*	*2.4*	*4.5*	0.5	0.3	0.3	0.1	0.3	0.0
	*JG30-KF-CM45*	0.6	0.6	0.8	1.7	1.2	*7.6*	*6.9*	*8.3*	0.9	1.2	1.7	1.6	*2.9*	2.0
Deinococcus-Thermus	*Trueperaceae*	0.3	0.0	0.0	0.0	0.0	1.2	0.9	0.9	0.2	0.1	*2.2*	0.0	1.0	0.1
Firmicutes	*Bacillaceae*	0.0	0.0	0.4	0.0	0.0	0.2	0.2	0.2	*6.8*	*5.3*	*30.8*	*4.4*	*8.6*	*4.5*
	*Paenibacillaceae*	0.0	0.0	0.0	0.0	0.0	0.0	0.0	0.0	1.6	*2.1*	*3.3*	0.8	1.5	1.0
	*Sporolactobacillaceae*	0.0	0.0	0.0	0.0	0.0	0.0	0.0	0.0	0.1	*10.9*	0.4	0.0	0.0	0.0
Gemmatimonadetes	*Longimicrobiaceae*	0.6	0.3	1.6	0.5	0.2	*5.0*	*4.2*	*3.8*	0.4	0.7	*2.9*	0.4	1.2	1.1
Planctomycetes	*WD2101*	0.4	0.0	0.0	*3.1*	1.0	0.7	0.3	0.5	*2.3*	*2.7*	1.1	1.7	1.9	*2.9*
Proteobacteria	*Sphingomonadaceae*	0.6	0.5	0.2	1.5	0.8	*2.5*	*3.8*	*3.3*	*3.5*	*3.0*	*2.0*	*2.1*	*2.5*	1.3
	*Burkholderiaceae*	*9.6*	*5.5*	*9.6*	1.0	1.6	*2.0*	1.7	*4.2*	0.6	0.7	1.2	0.5	0.7	0.7
	*Nitrosococcaceae*	0.0	0.0	0.0	*7.0*	1.8	0.0	0.0	0.0	1.7	0.6	0.1	0.4	0.2	1.1
Verrucomicrobia	*Chthoniobacteraceae*	0.0	0.0	0.0	0.4	0.2	0.0	0.0	0.0	*2.5*	*2.5*	0.3	*3.0*	1.9	*3.9*

ANCOM results suggested that genus *Streptomyces* (Actinobacteria) was significantly different (*W* = 703, *p* < 0.05) between the hyperarid core and transition zone. Characteristic known genera in the hyperarid core were *Thermus* (Deinococcus-Thermus), *Escherichia-Shigella* (Gammaproteobacteria), and *Pseudomonas* (Gammaproteobacteria), while characteristic known genera in the transition zone were *Rubrobacter* (Actinobacteria), uncultured Gammaproteobacteria, *Bacillus* (Firmicutes), and *Solirubrobacter* (Actinobacteria).

Hierarchical clustering of species indicated that the three pits within each individual AT-17 site generally had 40%–75% similarity (Fig. 4c). The hyperarid microbiome in MES was most similar to the most northern transition site TZ-0, which is in agreement with the statistical classification based on soil fundamental physicochemical parameters [41].

Prior to the marker gene pathway inference, 17 out of 4250 OTUs with NSTI more than 2 were excluded. By Nucleotide BLAST searching, these 17 excluded OTUs were all uncultured bacterium clones when *E* value < 10^−50^ and percent identity > 80%; 4 of them also closely related to some Candidatus phylum groups, i.e., Parcubacteria, Nomurabacteria, Uhrbacteria, and Adlerbacteria, respectively. Because of the microbiomic similarity, the metagenome function of MES microbiomes was comparable to the southern transition sites (Fig. 4b). However, the hyperarid site MES had fewer proportions of amine and polyamine biosynthesis, aminoacyl-tRNA charging, detoxification, glycan biosynthesis and degradation, nucleoside and nucleotide degradation, polymeric compound degradation, and secondary metabolite degradation.

Various adaptation pathways coupled with environmental stress reactions (including regulations of desiccation, irradiation, salinity, and osmotic pressure) and salt/small organic consumptions were predicted based on feature sequences (Figure S3). Compared to the more southern transition sites, MES and TZ-0 had slightly lower proportions of pathways coupled with stress reactions but higher proportions of nitrate and sulfate utilization pathways (Table 5).

**Table 5:** Percentages (means ± standard errors) of predicted metabolic pathways related to stressor responses from Atacama soils in the hyperarid core and the transition zone sampled in 2017

Stressor response pathways (%)	AT-17 MES	AT-17 TZ-0	AT-17 TZ-4	AT-17 TZ-5	AT-17 TZ-6
Desiccation/dehydration	3.35 ± 0.18	3.36 ± 0.09	3.36 ± 0.03	3.53 ± 0.16	3.51 ± 0.03
Ultraviolet/irradiation	3.01 ± 0.15	2.88 ± 0.28	3.32 ± 0.07	3.75 ± 0.11	3.44 ± 0.01
Salinity	1.40 ± 0.00	1.46 ± 0.05	1.29 ± 0.01	1.11 ± 0.05	1.20 ± 0.02
Simple organic molecule metabolisms	8.37 ± 0.11	8.47 ± 0.30	8.74 ± 0.17	9.68 ± 0.15	9.62 ± 0.25
Nitrate reduction/assimilation	2.79 ± 0.15	2.56 ± 0.06	2.71 ± 0.05	2.60 ± 0.03	2.60 ± 0.13
Sulfate reduction/assimilation	1.25 ± 0.02	1.26 ± 0.05	1.13 ± 0.02	1.11 ± 0.01	1.12 ± 0.02
Osmotic lysis	7.19 ± 0.16	7.32 ± 0.18	7.35 ± 0.05	7.38 ± 0.23	7.43 ± 0.09

## Discussion

### Effects of water and salt regulations on microbial growth

In the Atacama Desert, atmospherically derived nitrate and other oxyanion salts accumulate in the soil [44], leading to variable soil conductivity, high oxidation potential, and abiotic chemical decomposition of soil organic matter. Our results indicated that while precipitation was a constraint for microbiomes, other geochemical properties also affected the microbial community structure, especially salt availability. As the aridity increased toward the northern hyperarid desert, soluble compounds accumulated at the surface and shallow subsurface. On the other hand, increased precipitation in the southern transition zone restricted these accruals as more salts were delivered deeper, but it also simultaneously enhanced soil salt availability [41, 44].

We found that the viable and total cell counts were remarkably lower in PONR and Yungay, the two hyperarid sites that received massive rainfall before sampling, than the other sites (Fig. 2a and Table S3). However, microbial viability in Atacama soils was variable, with the lowest in the southern desert sites TZ-4 to TZ-6, which was opposite to a previous study before the recent rainfall events [45]. This increased viability in the hyperarid region could be a consequence of the loss of non-viable cells as an additional implication of the rainfall discharge effects [46, 47]. Across the AT-17 sampling transect, the percentage of viable microbial cells decreased from ≤ 89% in northern hyperarid soils to 39% in southern transitional soils (Table S3), similar to other extreme environments [48–50] and lower than typical soils usually with more than 90% viable cells [51, 52].

The microbial cultivation experiments (without any amendments) demonstrated that the CFUs from AT-17 samples increased up to 4 orders of magnitude from the hyperarid sites to transition sites (Fig. 2b, c, d, e and Table S4). The reasons for the difference between viable cell counts and culturable microbial colony numbers might be that (1) more unculturable species lived in the hyperarid core [53, 54] and that (2) most of the hyperarid microbiomes were metabolically inactive [2, 4, 45, 55]. Since the Actinobacteria-dominated microbial community structure did not alter much between the hyperarid AT-17 and transition AT-17 sites (Fig. 4a), the latter hypothesis might play a more important role in the culturable CFUs. The agar-cultured bacteria from surface samples collected within the Atacama Desert were limited in diversity, and the majority were members of Actinobacteria and Firmicutes. A small amount of Proteobacteria and Bacteroidetes has also been recovered [2, 42]. More specifically, previously identified culturable bacteria belonged to *Geodermatophilaceae*, *Sphingomonas*, *Bacillus*, *Arthrobacter*, *Brevibacillus*, *Kocuria*, *Cellulomonas*, *Hymenobacter*, *Asticcacaulis*, *Mesorhizobium*, *Bradyrhizobium*, *Afipia*, *Alphaproteobacteria*, and *Betaproteobacteria* [21, 56]. On our agar plates, these bacterial taxa acted as representatives of the whole microbial community from their sampled sites. Thus, the manner of growth and the change in CFUs of our cultivation experiments primarily reflected the preferences of bacteria within these taxa.

The water amendment experiments suggested that active culturable microorganisms were not impaired by rainfall that was less than 1 mm per day, as the volumes of water we added to these agar plates. Although the proliferation of some of these microbes manifested a decreasing trend after the abrupt water wash, more microbes benefited from the addition of water (Table 1 and Table S4). However, when precipitation reached as high as the unprecedented rainfall event (19.6 mm) in a few days, massive water input could dissolve soluble salts and concentrated them down to more than 20-cm depth [57, 58]. Amendments with excessive dissolved sodium chloride and sodium carbonate inhibited the growth of active microorganisms on agar plates; amendments with excessive dissolved sodium sulfate and sodium acetate made no difference in microbial growth; only excessive sodium l-lactate amendments promoted the growth of active culturable microbes (Table 2 and Table S4). Unfortunately, since organic C salts were limited in the oxidizing natural environment of the Atacama Desert [2, 59], the dissolved material and the extreme water addition might only harm the indigenous microbial communities [6, 10], at least in the short term.

In comparison with the results of microbial cultures, the negative correlations between TOC (an approximation of all grain-bound biomass) [21, 41, 60] and sediment sodium/chloride within the hyperarid core (Fig. 3a and b) were consistent with the negative effects of sodium chloride and some other sodium salts on microbial proliferation (Table 2 and Table S4) [42]. However, TOC content in the hyperarid core was positively associated with water-soluble chloride (Fig. 3c). This inconsistency of the relationships between microbial biomass and chloride in different forms supported our hypotheses that the chloride mineral halite was highly hygroscopic, and the deliquescence process within chloride minerals provided moisture to endolithic microbial communities [61, 62]. The soluble chloride concentration indicated the portion of chloride that deliquesced. The positive correlation between TOC and soluble chloride reflects the beneficial effect of salt deliquescence on microbial biomass but not a direct benefit from chloride ions.

Furthermore, water-soluble sulfate also contributed to microbial biomass as a long-term outcome (Fig. 3d), which disagreed with salt amendment experiments (Table 2 and Table S4). Since sulfate in the Atacama Desert was primarily formed in the hydrated form gypsum (Fig. 1c) [63], more sulfate indicated that larger proportions of gypsum could provide crystallization water to nearby microorganisms [36, 64]. Additionally, some microorganisms could reduce sulfate for energy production and sulfur assimilation (Figure S3 and Table 5) [65, 66]. Therefore, higher sulfate concentrations could both elevate the moisture in a microhabitat and supply nutrients for microbial life.

### Microbial communities and metabolic functions along the latitudinal aridity gradient

After the heavy rainfall event in June 2017, soluble salts at the shallow subsurface of soils were dissolved and transported to depth. For example, the proportions of brushite and gypsum which contain crystallized water molecules increased remarkably in PONR and Yungay where the recent heavy rainfall had the largest influence (Fig. 1c and Figure S1) compared to minerals before rainfall [67], and chloride minerals became undetectable in all AT-17 sites. Adequate metagenome failed to be extracted from PONR and Yungay soils within the hyperarid area. These two sampling sites had undertaken the most amount of precipitation 6 months before our sampling. Despite more culturable colonies, the trypan blue assay-determined viable and total cell counts of PONR and Yungay were remarkably lower than the other sites, including MES. Additionally, Azua-Bustos et al. (2018) found that excessive rainfall input could significantly diminish microbial communities in the Atacama [6]. Given the water amendment cell culture experiments in this study, water addition probably raises the growth rate of culturable species only. This result also indicated that the extracted metagenomic DNA was contributed more by unculturable extremophilic species.

Although the diversity of unknown phyla could be as high as 40% [3], the abundance of these species was relatively low (Fig. 4a). Soils of the southern arid desert were generally homogeneous in microbial composition and dominated (> 2%) by xerotolerant, halotolerant, and radioresistant Actinobacteria, Chloroflexi, Proteobacteria, Firmicutes, Bacteroidetes, Gemmatimonadetes, Planctomycetes, and Acidobacteria (Fig. 4a), phyla commonly detected in arid desert environments [68]. MES of lower relative humidity than the hyperarid Yungay region [31] possessed 16% more Actinobacteria percentage than southern arid sites (Fig. 4a). However, compared to Yungay, MES had higher microbial abundance (Fig. 2a), which might result from proper but not overwhelming rainfall input. Given the 40% similarity between MES and TZ-0 species (Fig. 4c), the structure of microbial communities in the hyperarid region largely shifted toward a pattern that is more common in the southern transition zone (Fig. 4c), to one dominated by Actinobacteria, Proteobacteria, and Chloroflexi (Fig. 4a). This shift indicated the occurrence of a water-driven microbial community perturbation and reorganization in the hyperarid core. As for predicted metabolisms, MES microorganisms had lower proportions of degradation pathways of amines, polyamines, carboxylates, polymeric compounds, and secondary metabolites to save essential organic sources for survival (Fig. 4b).

If the microbial community composition in the pre-rainfall MES was assumed to be similar to the nearby northern hyperarid site KM40 sampled in 2012 [45], these pre-rainfall hyperarid soils were shown to be predominantly composed of Deinococcus-Thermus and Aquificae as previously reported in Shirey (2013), and other characteristic bacterial phyla included Acetothermia, Armatimonadetes, Hydrothermae, and Thermotogae, bacteria within which generally adapted to nutrient-depleted and high-temperature conditions. Actinobacteria that lived in the pre-rainfall hyperarid core of the Atacama Desert before rainfall were extremely low [45]. Besides the long-term hyperaridity, the deficiency of extractable Actinobacteria before rainfall may be also caused by incomplete extraction: some Actinobacteria such as its thermoalkaliphilic families [69] *Geodermatophilaceae*, *Nocardioidaceae*, *Pseudonocardiaceae*, *Rubrobacteriaceae*, and *Solirubrobacteraceae* (Table 4) could form spores that were difficult to break down during DNA extraction to counterattack ultraviolet radiation and dehydration [70]. Analogous to Actinobacteria, the decreased proportions of Firmicutes (*Bacillaceae*, *Paenibacillaceae*, and *Sporolactobacillaceae*) (Table 4) in the hyperarid core after the heavy rainfall might also be a result of their sporulation [71, 72]. *Chthoniobacteraceae* of Verrucomicrobia appeared only in the two most humid sampling sites, as they generally metabolize organic carbon from plants [73].

Deinococcus-Thermus bacteria are well-known extremophiles that have previously been detected in calcites, halites, microbialites, and gypsum evaporites of the Atacama Desert. After the heavy rain, within the phylum of Deinococcus-Thermus, dominance shifted from of the order Thermales [45], who were mostly thermoresistant, to a dominance of the order Deinococcales, who were both thermoresistant and radioresistant [74, 75]. This shift might be triggered by the negative facet of water-driven microbial metabolic activation: since water revived dormant microbes, these microbes disarmed sporulation and confronted the harsh ambient conditions, especially ionizing irradiation [7, 8]. Therefore, the Deinococcus-Thermus population shifted to a more Deinococcales-dominant structure.

On Mars, the nocturnal availability of a thin layer of liquid water at the subsurface [27, 76–78] can nourish indigenous Martian “microbes” and allow them to slowly thrive with a small amount of moisture daily [7]. The cycle of desiccation and rewetting facilitates dead microbial decomposition, releasing intracellular organics and soil organic matters [8], which also remobilizes nutrients for live microorganisms that are activated from dormancy by liquid water. However, most microorganisms were not culturable (staying dormant or dead) when exposed to brine [6]. Thus, our findings suggested searching for feasibly detectable Martian “life” during the daytime after the evaporation of the nighttime film of liquid brine. Confronting the benefits and challenges brought by the water and salt additions as the aftermath of rewetting, microbial communities in the hyperarid region could likely shift to a structure that is common under more humid conditions in accordance with this study. We therefore infer similar events that may have happened on the wetter early Mars, as well as during the drying-rewetting cycle on the more recent dry Mars.

## Supplementary information

ESM 1 (DOCX 794 kb)
