# Supplementary material for: Spatial Variability of Microbial Communities and Salt Distributions Across a Latitudinal Aridity Gradient in the Atacama Desert
Source: Microb Ecol. 2021 Jan 13;82(2):442–58. doi: 10.1007/s00248-020-01672-w (PMC8384830; doi:10.1007/s00248-020-01672-w)
Supplement: Supplementary file 1 — (DOCX 794 kb) [file 248_2020_1672_MOESM1_ESM.docx]

**Supplementary materials**

| 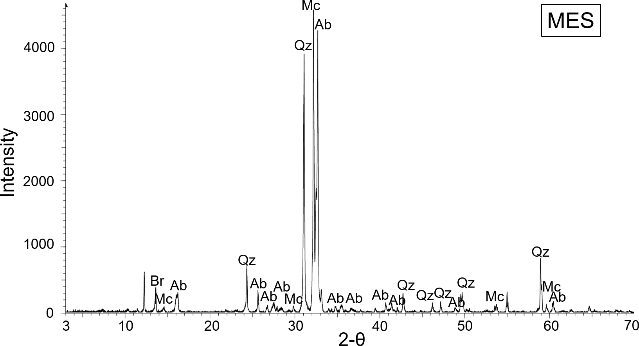  (**a**) | 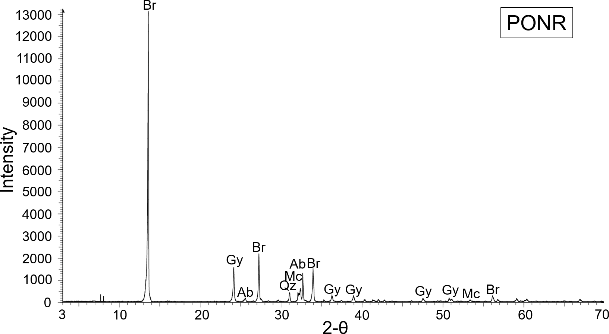  (**b**) |
| --- | --- |
| 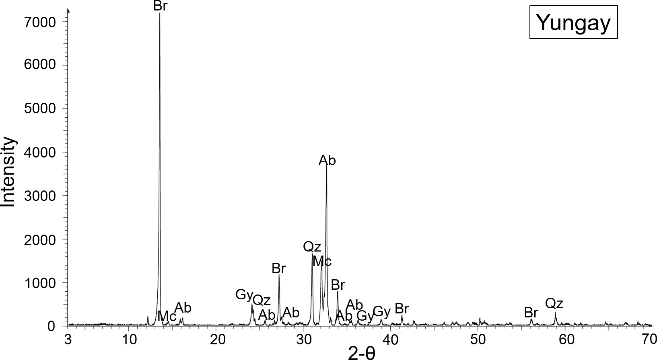  (**c**) | 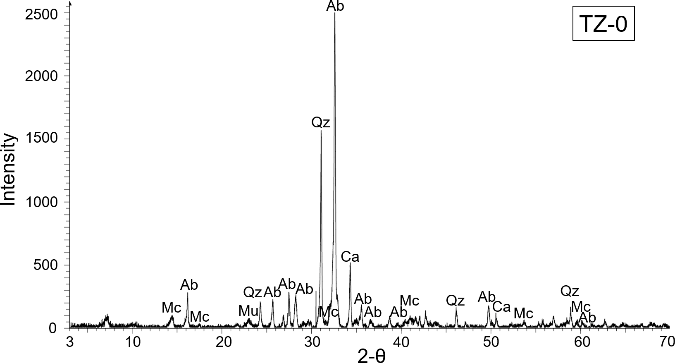  (**d**) |
| 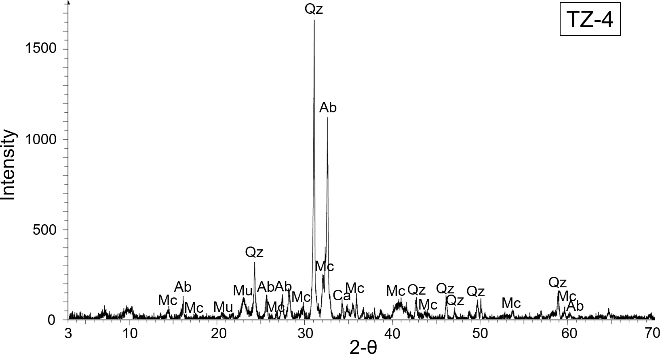  (**e**) | 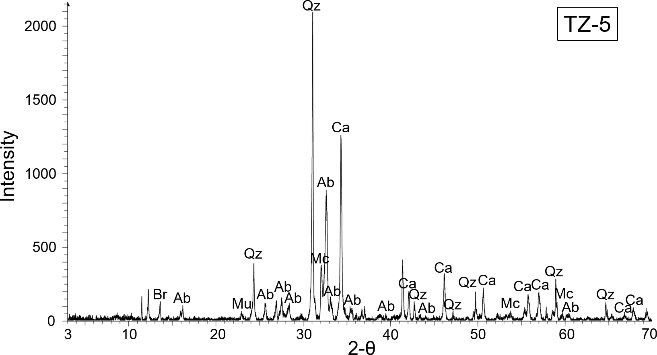  (**f**) |
| 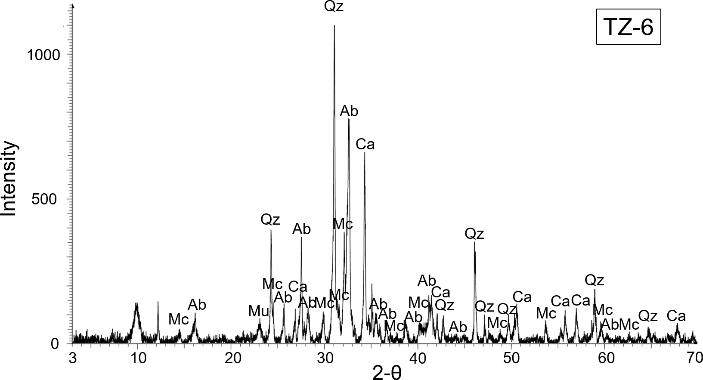  (**g**) |  |

**Figure S1.** Mineral compositions of AT-17 soils determined X-ray diffraction (XRD) analysis. (Qz, quartz; Ab, albite; Br, brushite; Gy, gypsum; Mu, muscovite; Ca, calcite; Mc, microcline)


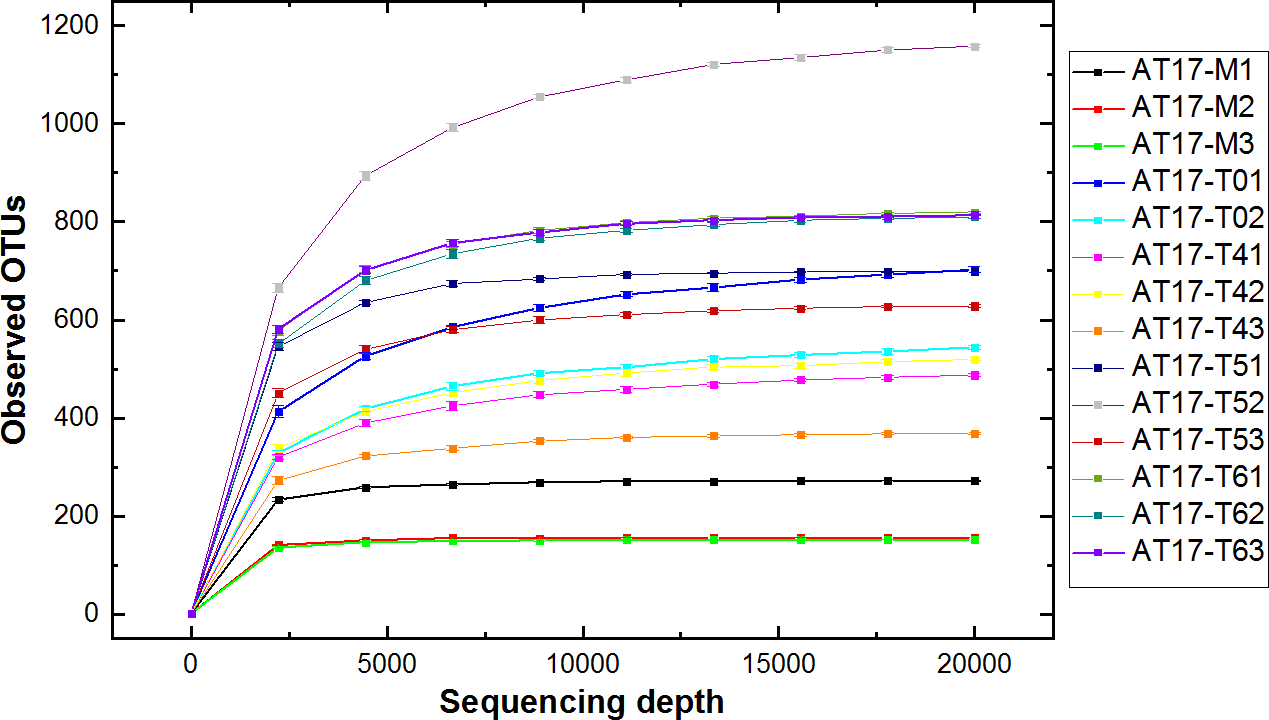


**Figure S2.** Observed OTUs richness as a function of the number of sequences per sample at 20,000 sampling depth. An OTU definition of sequence homology at or above 99%. Error bars of rarefaction analysis were shown. Abbreviations as in Table 3.


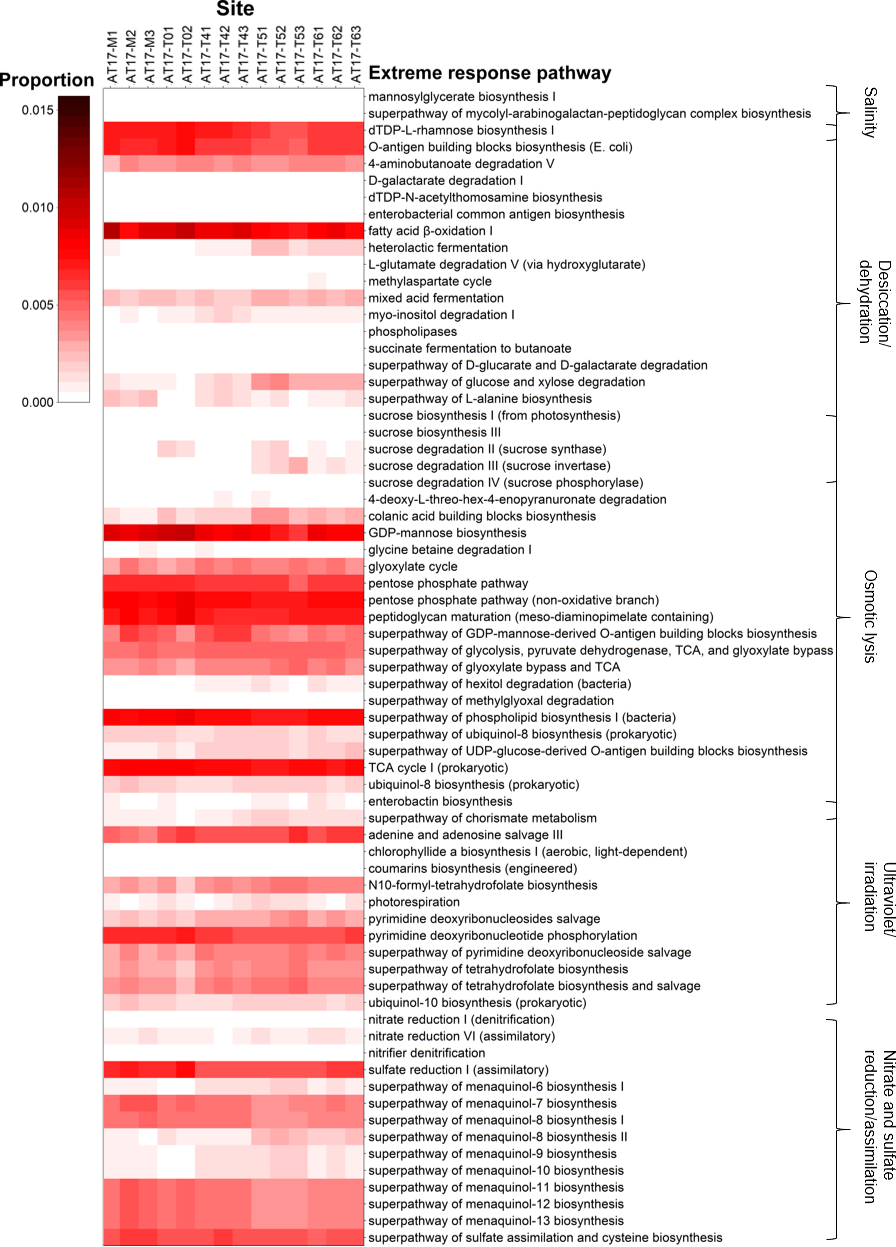


**Figure S3.** Relative abundance of microbial functional pathways that are associated with stressor responses from Atacama soil samples across a precipitation gradient collected in 2017.

**Table S1.** Geographic coordinates and altitudes of AT-17 sampling sites and the daily precipitation during the June 6-7 2017 heavy rainfall (modeled by <https://www.ventusky.com/>) [41,45].

| **Site** | **Latitude (°S)** | **Longitude (°W)** | **Altitude (m)** | **Precipitation (mm/day)** |
| --- | --- | --- | --- | --- |
| AT-17 MES | 22.2641 | 69.7243 | 1493 | 3.3 |
| AT-17 PONR | 23.0726 | 69.5892 | 1493 | 10.1 |
| AT-17 Yungay | 24.0884 | 69.9945 | 1007 | 13.4 |
| AT-17 TZ-0 | 26.3222 | 70.0128 | 1106 | 2.9 |
| AT-17 TZ-4 | 27.0565 | 69.9228 | 1658 | 3.9 |
| AT-17 TZ-5 | 27.6051 | 70.4458 | 588 | 0.4 |
| AT-17 TZ-6 | 28.4100 | 70.7270 | 658 | 0.1 |

**Table S2.** Concentrations (means ± standard errors) of soluble chloride, nitrate, and sulfate in AT-17 samples.

| **Sample** | **Cl^-^ (ppm)** | **NO_3_^-^ (ppm)** | **SO_4_^2-^ (ppm)** |
| --- | --- | --- | --- |
| MES pit 1 | 7.5±0.1 | 35.7±0.3 | 260.4±3.2 |
| MES pit 2 | 71.4±1.2 | 238.4±3.6 | 4948.0±60.5 |
| MES pit 3 | 8.5±0.1 | 52.1±0.2 | 5865.3±24.6 |
| PONR pit 1 | 54.3±1.6 | 147.5±2.5 | 14108.4±333.1 |
| PONR pit 2 | 226.7±22.6 | 362.0±39.1 | 19520.1±3172.1 |
| PONR pit 3 | 65.5±2.0 | 157.7±2.6 | 16093.5±333.0 |
| Yungay pit 1 | 16.4±0.9 | 23.8±0.1 | 15840.4±248.4 |
| Yungay pit 2 | 14.0±0.2 | 19.3±1.0 | 16941.8±1398.0 |
| Yungay pit 3 | 11.5±0.8 | 15.5±0.3 | 15672.4±190.0 |
| TZ-0 pit 1 | 7.0±0.1 | 1.9±0.0 | 16.7±0.3 |
| TZ-0 pit 2 | 4.1±0.1 | 1.4±0.0 | 25.7±0.4 |
| TZ-0 pit 3 | 6.2±0.1 | 2.5±0.0 | 28.2±0.3 |
| TZ-4 pit 1 | 3029.6±27.8 | 3002.3±26.8 | 165.9±2.2 |
| TZ-4 pit 2 | 5040.2±48.6 | 7448.2±66.7 | 338.9±3.5 |
| TZ-4 pit 3 | 2615.0±26.5 | 4456.0±43.6 | 1212.0±13.5 |
| TZ-5 pit 1 | 38.2±0.4 | 3.0±0.1 | 13.7±0.1 |
| TZ-5 pit 2 | 328.9±2.5 | 6.0±0.1 | 1606.4±10.7 |
| TZ-5 pit 3 | 3132.1±19.9 | 20.9±0.2 | 3564.0±18.9 |
| TZ-6 pit 1 | 113.9±0.8 | 11.7±0.2 | 95.9±0.8 |
| TZ-6 pit 2 | 2981.5±18.5 | 140.6±1.3 | 1660.8±10.3 |
| TZ-6 pit 3 | 1269.0±9.8 | 82.4±0.8 | 489.4±3.9 |

**Table S3.** Bacterial abundance and viability within AT-17 soil determined by trypan blue staining assay analyses.

| **Sample** | **log(viable cells/g)** | **log(non-viable cells/g)** | **log(total cells/g)** | **viable : total (cells/cells)** |
| --- | --- | --- | --- | --- |
| MES | 6.67 | 5.76 | 6.72 | 0.89 |
| PONR | 6.25 | 5.67 | 6.35 | 0.79 |
| Yungay | 6.32 | 5.78 | 6.43 | 0.78 |
| TZ-0 | 6.77 | 5.99 | 6.84 | 0.86 |
| TZ-4 | 6.53 | 6.43 | 6.78 | 0.55 |
| TZ-5 | 6.26 | 6.39 | 6.66 | 0.39 |
| TZ-6 | 6.70 | 6.37 | 6.90 | 0.64 |

**Table S4.** Colony forming units (CFUs) of AT-17 soil samples on ultrapure agarose, tryptic soy agar, LB agar, and plate count agar plates without amendments, amended with water, and amended with 10% sodium chloride, 10% sodium sulfate, 10% sodium carbonate, 10% sodium acetate, and 10% sodium L-lactate.

| **Type of culture plate** | **Amendment** | **MES** | **PONR** | **Yungay** | **TZ-0** | **TZ-4** | **TZ-5** | **TZ-6** |
| --- | --- | --- | --- | --- | --- | --- | --- | --- |
| Ultrapure agarose | None | 15 | 186 | 55 | 770 | 5.73×10^3^ | 968 | 5.29×10^3^ |
|  | 1.5 mL H_2_O | 0 | 207 | 196 | 100 | 2.71×10^3^ | 69 | 437 |
|  | 3 mL H_2_O | 0 | 481 | 134 | 195 | 3.07×10^3^ | 104 | 1.25×10^3^ |
|  | 4.5 mL H_2_O | 0 | 15 | 106 | 111 | 3.57×10^3^ | 87 | 667 |
|  | Chloride | 0 | 2 | 459 | 295 | 3248 | 4.09×10^3^ | 2.90×10^3^ |
|  | Sulfate | 0 | 0 | 2 | 27 | 1595 | 87 | 773 |
|  | Carbonate | 0 | 5 | 104 | 0 | 1175 | 87 | 3.50×10^3^ |
|  | Acetate | 0 | 0 | 247 | 488 | 406 | 696 | 1.51×10^3^ |
|  | L-lactate | 218 | 1.08×10^3^ | 503 | 1.12×10^3^ | 116 | 3.68×10^3^ | 1.02×10^3^ |
|  |  |  |  |  |  |  |  |  |
| Tryptic soy agar | None | 37 | 53 | 169 | 514 | 1.60×10^5^ | 1.33×10^5^ | 3.21×10^5^ |
|  | 1.5 mL H_2_O | 523 | 140 | 44 | 297 | 1.09×10^5^ | 5.75×10^4^ | 2.21×10^5^ |
|  | 3 mL H_2_O | 41 | 35 | 6.29×10^3^ | 288 | 8.00×10^4^ | 1.20×10^5^ | 4.29×10^5^ |
|  | 4.5 mL H_2_O | 5.76×10^3^ | 3.39×10^3^ | 1.14×10^4^ | 312 | 1.65×10^6^ | 5.22×10^4^ | 5.09×10^5^ |
|  | Chloride | 7 | 12 | 3.6×10^4^ | 181 | 4.21×10^4^ | 8.70×10^5^ | 5.26×10^5^ |
|  | Sulfate | 22 | 5 | 1.28×10^4^ | 1.18×10^3^ | 2.80×10^5^ | 3.47×10^5^ | 5.16×10^5^ |
|  | Carbonate | 394 | 10 | 2.69×10^4^ | 7 | 298 | 5.44×10^4^ | 3.68×10^5^ |
|  | Acetate | 2 | 2 | 92 | 268 | 5.08×10^5^ | 1.45×10^5^ | 1.11×10^6^ |
|  | L-lactate | 2.19×10^4^ | 58 | 6.94×10^3^ | 575 | 1.77×10^6^ | 8.34×10^4^ | 4.77×10^5^ |
|  |  |  |  |  |  |  |  |  |
| LB agar | None | 17 | 28 | 33 | 91 | 2.39×10^5^ | 2.95×10^3^ | 5.41×10^4^ |
|  | 1.5 mL H_2_O | 71 | 54 | 13 | 140 | 8.18×10^3^ | 1.81×10^3^ | 1.65×10^5^ |
|  | 3 mL H_2_O | 37 | 93 | 7.51×10^3^ | 241 | 2.67×10^4^ | 4.93×10^3^ | 1.14×10^5^ |
|  | 4.5 mL H_2_O | 7 | 7.98×10^3^ | 3.71×10^3^ | 491 | 1.23×10^6^ | 1.17×10^3^ | 1.54×10^5^ |
|  | Chloride | 0 | 2 | 219 | 135 | 3.03×10^3^ | 986 | 1.19×10^5^ |
|  | Sulfate | 22 | 2 | 145 | 858 | 9.37×10^3^ | 2.96×10^3^ | 3.71×10^5^ |
|  | Carbonate | 2.68×10^3^ | 15 | 53 | 0 | 305 | 1.96×10^3^ | 4.49×10^3^ |
|  | Acetate | 10 | 5 | 243 | 358 | 5.44×10^5^ | 4.35×10^3^ | 6.01×10^5^ |
|  | L-lactate | 162 | 184 | 1.10×10^4^ | 1.08×10^3^ | 3.28×10^6^ | 6.21×10^3^ | 4.27×10^5^ |
|  |  |  |  |  |  |  |  |  |
| Plate count agar | None | 167 | 190 | 8.06×10^3^ | 1.40×10^3^ | 6.89×10^6^ | 1.82×10^4^ | 1.25×10^6^ |
|  | 1.5 mL H_2_O | 744 | 8.43×10^3^ | 8.43×10^3^ | 311 | 3.22×10^6^ | 5.87×10^4^ | 6.44×10^6^ |
|  | 3 mL H_2_O | 2.09×10^3^ | 254 | 4.55×10^4^ | 243 | 2.67×10^6^ | 2.60×10^4^ | 5.93×10^5^ |
|  | 4.5 mL H_2_O | 1.36×10^3^ | 8.56×10^4^ | 6.96×10^4^ | 638 | 1.33×10^7^ | 1.25×10^5^ | 4.12×10^6^ |
|  | Chloride | 2 | 53 | 29 | 237 | 1.89×10^6^ | 5.80×10^4^ | 5.68×10^5^ |
|  | Sulfate | 94 | 880 | 0 | 9.18×10^3^ | 6.09×10^6^ | 5.80×10^4^ | 4.18×10^5^ |
|  | Carbonate | 1.61×10^3^ | 176 | 174 | 53 | 4.35×10^3^ | 6.38×10^4^ | 1.74×10^5^ |
|  | Acetate | 2 | 19 | 135 | 193 | 2.67×10^6^ | 2.32×10^4^ | 1.33×10^6^ |
|  | L-lactate | 2.93×10^3^ | 297 | 6.82×10^4^ | 2.84×10^3^ | 2.78×10^7^ | 3.97×10^5^ | 2.60×10^6^ |
